# Correction to “The Impact of Endoscopic Ultrasound and Multidisciplinary Team Evaluation on the Management of Pancreatic Cystic Lesions”

**DOI:** 10.1002/ueg2.70234

**Published:** 2026-06-16

Dbouk, Mohamad, Osama Altayar, Mark J. Hoegger, et al. 2026. “The Impact of Endoscopic Ultrasound and Multidisciplinary Team Evaluation on the Management of Pancreatic Cystic Lesions,” *United European Gastroenterology Journal*: e70212. https://doi.org/10.1002/ueg2.70212.

In Tables 2 and 4, the header “Group 1 (EUS)” is incorrect. This should have read as “Group 1 (No EUS).” The header “Group 2 (No EUS)” is incorrect. This should have read as “Group 2 (EUS).” In the legend of Figure 2, “(A) MDT Group 1 (EUS)” is incorrect, this should have read as “(A) MDT Group 1 (No EUS).” Additionally, “(B) Group 2 (No EUS)” is incorrect, this should have read as “(B) Group 2 (EUS).”

**TABLE 2:** Performance of MDT groups for PCL management.

	Group 1 (No EUS)			Group 2 (EUS)			*p*‐value (1 vs. 2)	Group 3 (Unblinded to EUS)			*p*‐value (1 vs. 3)	*p*‐value (2 vs. 3)
Variable	Sensitivity (CI)	Specificity (CI)	AUC (CI)	Sensitivity (CI)	Specificity (CI)	AUC (CI)		Sensitivity (CI)	Specificity (CI)	AUC (CI)		
Identification of mucinous cyst	91.43 (82.3–96.8)	20 (2.5–55.6)	0.557 (0.442–0.668)	97.14 (90.1–99.7)	70 (34.8–93.3)	0.836 (0.736–0.909)	**0.04**	97.14 (90.1–99.7)	10 (0.3–44.5)	0.536 (0.421–0.648)	0.81	**0.0003**
Identification of advanced neoplasia	62.5 (45.8–77.3)	75 (58.8–87.3)	0.688 (0.574–0.787)	80 (64.4–90.9)	82.5 (67.2–92.7)	0.812 (0.710–0.889)	**0.029**	75 (58.8–87.3)	82.5 (67.2–92.7)	0.788 (0.682–0.871)	**0.009**	0.59
Appropriate recommendation for surgery	60 (43.3–75.1)	75 (58.8–87.3)	0.675 (0.561–0.776)	85 (70.2–94.3)	85 (70.2–94.3)	0.85 (0.753–0.92)	**0.0016**	72.5 (56.1–85.4)	80 (64.4–90.9)	0.763 (0.654–0.851)	**0.0311**	**0.0485**

Abbreviations: Advanced Neoplasia = high‐grade dysplasia or invasive cancer, AUC = area under curve, CI = confidence interval, EUS = endoscopic ultrasound, MDT = multi‐disciplinary team.

**TABLE 4:** Interobserver agreement of MDT between centers.

Variable	Kappa (CI)		
	Group 1 (No EUS)	Group 2 (EUS)	Group 3 (Unblinded to EUS)
Identification of mucinous cyst	0.444 (−0.01–0.9)	0.62 (0.23–1)	0.655 (0.02–1)
Identification of advanced neoplasia	0.35 (0.068–0.63)	0.75 (0.54–0.95)	0.46 (0.21–0.71)
Resection recommendation (Referral to surgery)	0.5 (0.24–0.75)	0.9 (0.76–1)	0.56 (0.32–0.79)

Abbreviations: Advanced Neoplasia = high‐grade dysplasia of invasive cancer, CI = confidence interval, PCL = pancreatic cystic lesion.

**FIGURE 2:**
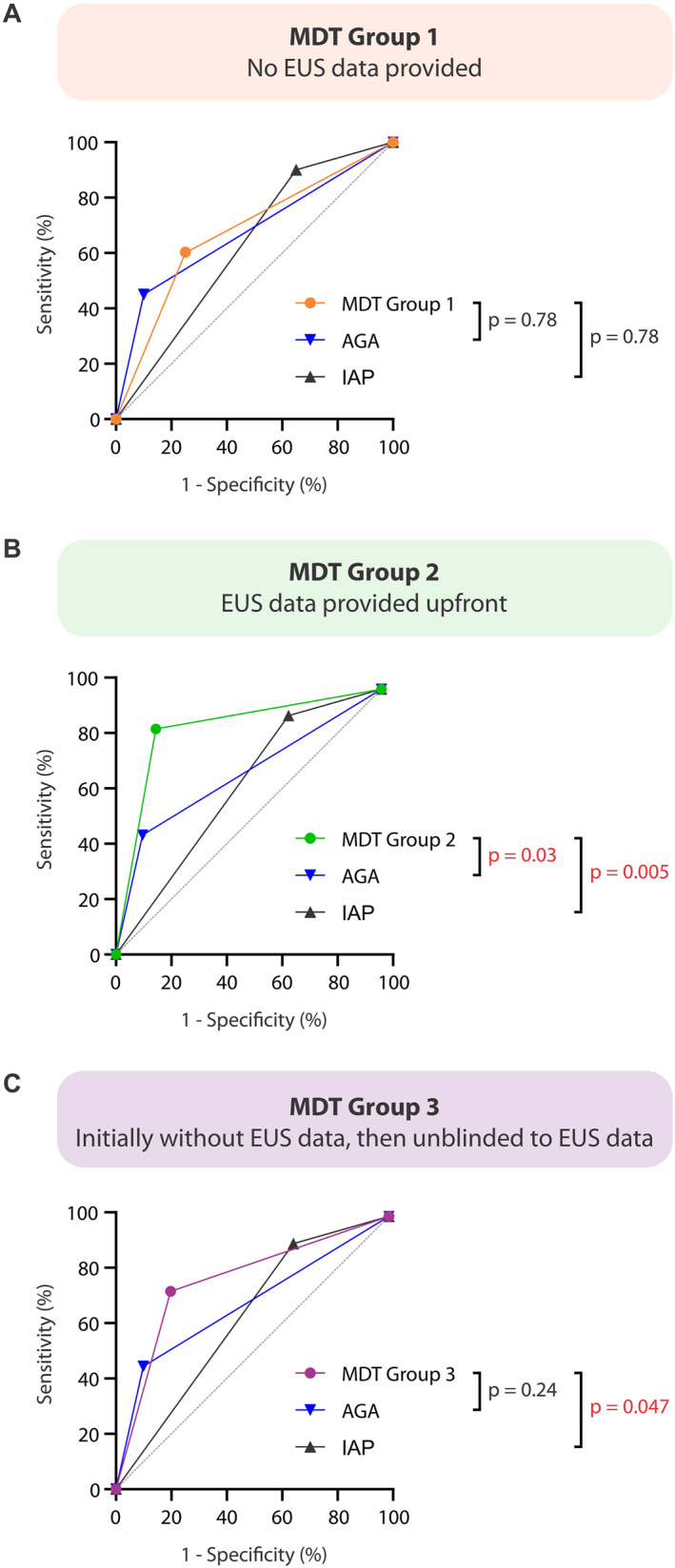
Performance of mock MDT groups compared to AGA and IAP clinical guidelines for appropriate surgical referral of PCL to surgery. (A) MDT Group 1 (No EUS) (AUC 0.675 (CI 0.56–0.79)) performed similar to AGA (AUC 0.67 (CI 0.51–0.84); *p* = 0.78) and IAP guidelines (AUC 0.62 (CI 0.45–0.80); *p* = 0.78). (B) Group 2 (EUS) (AUC 0.85 (CI 0.76–0.94)) performed significantly better than AGA (*p* = 0.03) and IAP (*p* = 0.005). (C) Group 3 (Unblinded to EUS) (AUC 0.76 (CI 0.65–0.87)) performed similar to AGA (*p* = 0.24) but better than IAP (*p* = 0.047).

We apologize for these errors.

